# A systematic review of the prevalence of Morquio A syndrome: challenges for study reporting in rare diseases

**DOI:** 10.1186/s13023-014-0173-x

**Published:** 2014-11-18

**Authors:** Regina M Leadley, Shona Lang, Kate Misso, Trudy Bekkering, Janine Ross, Takeyuki Akiyama, Michael Fietz, Roberto Giugliani, Chris J Hendriksz, Ngu Lock Hock, Jim McGill, Andrew Olaye, Mohit Jain, Jos Kleijnen

**Affiliations:** Kleijnen Systematic Reviews, Unit 6, Escrick Business Park, Escrick, York, YO19 6FD UK; BeSyReBekkering Systematic Reviews, Geel, Belgium; Japan society of patients and families with Mucopolysaccharidoses, 2-37-3, Minamisyojyaku, Suita-Shi, Osaka 564-0012 Japan; Department of Biochemical Genetics, SA Pathology (at WCH), 72 King William Road, North Adelaide, SA 5006 Australia; Chief of the Medical Genetics Service, Clinic Hospital of Porto, Porto Alegre, Rio Grande do Sul Brazil; Clinical lead- Adult Inherited Metabolic Disorders, Consultant Transitional Metabolic Medicine, Manchester Academic Health Science Centre, The Mark Holland Metabolic Unit, Salford Royal Foundation NHS Trust, Ladywell NW2- 2nd Floor Room 107, Salford, Manchester, M6 8H UK; Consultant Pediatrician and Clinical Geneticist, Clinical Genetics & Metabolic Department, Kuala Lumpur Hospital, Jalan Pahang, Kuala Lumpur, 50586 Malaysia; Metabolic physician and Clinical geneticist Department of Metabolic Medicine, Royal Children’s Hospital, Brisbane, Australia; Snr Market Access Manager EUMEA, BioMarin Europe Ltd, 164 Shaftesbury Ave, London, WC2H 8HL United Kingdom; Market Access & Public Policy EUMEA BioMarin Europe Ltd, 164 Shaftesbury Ave, London, WC2H 8HL United Kingdom; School for Public Health and Primary Care (CAPHRI), Maastricht University, Maastricht, Netherlands

**Keywords:** Mucopolysaccharidoses type A, Morquio A, MPS IVA, Epidemiology, Prevalence, Incidence

## Abstract

**Background:**

Morquio A (MPS IVA) is a rare disease characterised by a deficiency of N-acetylgalactosamine-6 sulfatase (GALNS) and presenting with short stature, abnormal gait, cervical spine instability and shortened lifespan.

**Purpose:**

To prepare a systematic review of the prevalence of Morquio A in multiple countries and suggest recommendations for reporting rare diseases.

**Methods:**

Medline, Medline In-Process, Medline Daily Update, Embase, Cochrane Database of Systematic Reviews, Cochrane Central Register of Controlled Trials, Database of Abstracts of Reviews of Effects, Health Technology Assessment Database and PROSPERO were searched from inception to October 2013 to identify relevant information on the epidemiology of Morquio A. Forty Patient Organisation Representatives (POR) and Key Opinion Leaders (KOL) across 24 countries were contacted for data. Observational studies were included and case reports were excluded. Searches were performed without date or language restriction. Two researchers independently screened and extracted data. Quality of study reporting was assessed using a checklist adapted from STROBE (STrengthening the Reporting of OBservational studies in Epidemiology). Point or birth prevalence was stratified according to diagnostic method and discussed narratively.

**Results:**

In total 9,074 records were retrieved from searching and 25 studies were included for data extraction. Twenty out of 40 KOL and POR responded (50%) and 9 provided data (23%). Point prevalence of Morquio A was 1 per 926,000 in Australia, 1 per 1,872,000 in Malaysia and 1 per 599,000 in UK and Morquio (unclassified) was 1 per 323, 000 in Denmark. Birth prevalence of Morquio A (using recommended diagnostic methods) ranged from 1 per 71,000 in UAE to 1 per 500,000 in Japan. All results were compromised by poor study reporting and internal validity.

**Conclusions:**

The review highlighted that there is a misunderstanding of the definitions for prevalence and incidence in the field; that studies were poorly reported (diagnostic methods and patient characteristics) and that no suitable quality assessment tool exists. Overestimation and underestimation of prevalence data can occur. Bespoke reporting guidelines and a quality assessment tool specifically for prevalence of rare diseases are recommended.

**Electronic supplementary material:**

The online version of this article (doi:10.1186/s13023-014-0173-x) contains supplementary material, which is available to authorized users.

## Background

The Mucopolysaccharidoses (MPS) are a group of inherited metabolic disorders caused by a deficiency or malfunctioning of lysosomal enzymes which are needed to break down complex carbohydrates known as mucopolysaccharides or glycosaminoglycans (GAGs). Accumulation of GAGs causes a cascade of events leading to the progressive damage of cells, tissue and organs. Morquio disease or Mucopolysaccharidosis Type IV (MPS IV) belongs to this group and has two sub-types, A and B. Type A is also known as Morquio A, GALNS deficiency, Galactosamine-6-sulfatase deficiency, N-acetylgalactosamine-6-sulfate sulfatase deficiency or more simply MPS IVA, while type B is known as Morquio B, beta-galactosidase deficiency or MPS IVB. They are often grouped together with other rare diseases under the umbrella of lysosomal storage disorders (LSDs). The Orpha number for Morquio A is ORPHA309297. Morquio A is characterised by a deficiency of N-acetylgalactosamine-6 sulfatase (GALNS) [1], which results in a build-up of chondroitin-6-sulfate (C6S) and keratan sulfate (KS) in many tissues and organs. This build up results in multi-systemic clinical impairments including musculoskeletal abnormalities, short stature, pulmonary and cardiac dysfunction, hearing loss and corneal clouding [2]. A recent report by Lavery and Hendriksz (2014) reported the average age of death for a Morquio A patient in the UK to be 25 years with respiratory failure as the main cause of death (63%) [3]. Morquio B results from a deficiency of beta-galactosidase activity and usually patients have a more attenuated phenotype compared to Morquio A [4,5]. It is important to mention that in many cases beta-galactosidase deficiency leads to accumulation of GM1 ganglioside, causing the neurodegenerative disease GM1 gangliosidosis (with early infantile, juvenile of adult phenotypes). To date, the full spectrum of Morquio B has not been well documented, with reports of patients with intermediate phenotype between Morquio B and GM1 gangliosidosis [6,7]. The clinical symptoms for Morquio were first described in 1929 [8], but it was not until the 1960s that elevated levels of GAGs in the urine of patients were discovered and the term MPS IV was used (reviewed in Hendriksz et al. 2013 [9]). In 1976, Singh et al. found that the enzyme N-acetylgalactosamine-6 sulfatase was deficient in Morquio A and in the following year Morquio B identified as a separate entity [9].

Where Morquio A or B is clinically suspected, the recommended means of obtaining a diagnosis is by measuring the enzymatic activity of GALNS or beta-galactosidase respectively in either fibroblast or leukocyte samples [9-11]. Measurement of enzyme activity in dried blood spots (DBS) can be useful [12], but it is recommended that low activities should be confirmed in leucocytes as activity could be impaired by transportation and/or storage in sub-optimal conditions [8]. Additional biochemical investigation and/or genetic analysis should be performed to rule out potential false positives incurred by different diseases such as multiple sulfatase deficiency (MSD) or mucolipidosis II or III (ML II/III) where low GALNS activity may be present [10]. Other diagnostic methods, namely urinary KS identification by thin-layer chromatography or electrophoresis, or tandem mass spectrometry (TMS), or KS measurement in plasma or DBS can be used as screening methods. When KS detection is used as screening, the likelihood of false negatives becomes higher as patients age. One of the main sources of KS is cartilage, thus when bone growth plates close in patients reaching puberty the rate of KS accumulation slows and urinary excretion is substantially reduced and may even disappear [13]. Measurement of KS is not specific to Morquio A as patients with Morquio B and MSD also excrete increased amounts of KS [5,14]. Elevated KS has been reported in other MPS disorders and mucolipidoses [12]. KS can also be elevated in other LSDs such as Fucosidosis and GM1 gangliosidosis [15]. All diagnostics methods should be clearly reported and should include a description of how the samples were stored.

Molecular analysis can confirm biochemical analyses and more than 180 different mutations have been identified for the GALNS gene, but in isolation it can inconclusive or complicate the diagnostic process as common mutations have only been identified for specific ethnic sub-populations [9] and variants of unknown significance may be found. Genetic analysis of cases already diagnosed is useful to identify new mutations and also to identify carriers, being helpful for genetic counselling and prenatal diagnosis [10]. Treatment measures were largely palliative and focussed on alleviating organ specific complications [15] until specific enzyme replacement therapy (ERT) became approved [16].

Morquio A and B are both autosomal recessive inherited conditions which affect males and females equally and in most cases, both parents of an affected child are asymptomatic carriers of the disease. Morquio A is a rare condition and existing data on prevalence are scarce and variable. Reported estimates range from 1 per 76, 320 in Northern Ireland [17] to 1 per 641,178 in Western Australia [18]. Morquio B is even more rare and as a consequence, data on prevalence are even more scarce. It is important to consider factors such as consanguinity, migration and founders effect as they can have a profound effect on prevalence of genetic disorders. Up to date and accurate prevalence data are required to inform policy makers and indicate a need for development and direction of treatment. Currently it is unclear what the exact prevalence of Morquio A is and there are different methods for calculating prevalence and reporting data from rare diseases. The aim of the paper was to conduct a systematic review of the prevalence of Morquio A in multiple countries. In addition we discuss the challenges and limitations of completing a systematic review in the field of rare diseases.

## Methods

This systematic review was carried out in a transparent and reproducible nature as recommended by the Cochrane Collaboration [19] and the Centre for Reviews and Dissemination [20] and in accordance with the PRISMA statement [21].

### Inclusion criteria

We included observational studies that reported the prevalence or incidence of MPS IV, MPS IVA, Morquio A and over-arching terms such as Lysosomal storage disorders (including synonyms) from the following countries (without further limitations): Australia, Brazil, Canada, Colombia, Denmark, France, Germany, Italy, Japan, Malaysia, Mexico, Netherlands, Poland, Portugal, Qatar, Russia, Saudi Arabia, Spain, South Korea, Taiwan, Turkey, United Arab Emirates (UAE), United Kingdom (UK) and United States of America (USA). These countries were chosen on the basis of current known cases of Morquio A. Case series (from 1970) were included in the absence of prevalence or incidence data (if they reported newly diagnosed cases and a time period for data collection). Where Morquio or MPS IV was reported and no distinction was made between A and B, we categorised these data as Morquio (unclassified). Case reports were not included.

### Literature searches

Searches were carried out from inception to October 2013 in Medline; Medline In-Process & Other Non-Indexed Citations; Medline Daily Update; Embase; Cochrane Database of Systematic Reviews (CDSR); Cochrane Central Register of Controlled Trials (CENTRAL); Database of Abstracts of Reviews of Effects (DARE); Health Technology Assessment Database (HTA); and PROSPERO (International Prospective Register of Systematic Reviews) to identify relevant information on the epidemiology and prevalence of Morquio A. Only studies conducted in humans were sought. Searches were not limited by date (except for prior stated study design criteria), language or publication status (unpublished or published). An example of the search strategy is documented in Additional file 1. The main Embase strategy for each set of searches was independently peer reviewed by a second Information Specialist, using the CADTH checklist [22].

Supplementary searches were undertaken to identify completed and ongoing trials, on NIH ClinicalTrials.gov (http://www.clinicaltrials.gov); MetaRegister of Controlled Trials (mRCT); (http://www.controlled-trials.com) and WHO International Clinical Trials Registry Platform (ICTRP) (http://www.who.int/ictrp/en), from inception to October 2013. All identified references were imported into Endnote X4 software and duplicates were removed. The bibliographies of identified research and review articles were checked for additional relevant studies. Initial searches based on specific terms for Morquio A failed to retrieve some known studies; therefore broader terms for mucopolysaccharidoses and lysosomal storage disorders were employed.

### Contacting registries, patient organisations and key opinion leaders

For each included country, key opinion leaders and patient organisations were contacted where possible. Each person contacted was requested to provide information on: prevalence/incidence/birth rate/number of cases (including units of measure), definition of prevalence/incidence/birth rate/number of cases or method for calculation, time period over which data were gathered, geographical location, method/criteria for diagnosis of to identify relevant information on the epidemiology and prevalence of Morquio A, % male, age, weight, height, ethnicity and any additional comments.

### Methods of study selection, quality assessment and data extraction

Titles and abstracts were independently screened by two reviewers; full papers obtained for agreed relevant studies were again independently examined in detail to determine whether they met the inclusion criteria. All studies excluded based on reading the full paper were documented along with the reasons for exclusion. Quality of study reporting was independently assessed by two reviewers using a checklist we adapted from the STROBE (STrengthening the Reporting of OBservational studies in Epidemiology) statement for the reporting observational studies specifically for the review [23]. A guide to assessing the quality of study reporting (also developed for this review) is included in Additional file 2. The following information was extracted from studies: author, year, country, type of Morquio, study data collection period, study design, study setting, number lost to follow-up, diagnosis, method for genotyping, predefined inclusion and/or exclusion criteria, description of the participants included in the study, genotype, ethnic background, evidence of consanguinity, study aim and prevalence/incidence/birth rate/number of cases (including units of measure), definition of prevalence/incidence/birth rate/number of cases or method for calculation and reported study limitations. Data were extracted into specially designed extraction sheets in Microsoft Excel 2007 by one reviewer and checked by a second. Any discrepancies between reviewers were resolved through discussion or the intervention of a third reviewer.

### Analysis

To summarise data and compare the prevalence of Morquio A across countries, data were grouped according to the definition of point prevalence or birth prevalence. Given that these are inherited conditions, people cannot acquire them nor be cured, consequently “incidence” (number of new cases in a given time period) is a less helpful construct. If incidence was reported we analysed the definition to determine whether or not it was equivalent to a definition for point prevalence or birth prevalence. Those that were identical were included under a new prevalence definition. Only postnatal diagnoses or prenatal diagnoses that resulted in a live birth were included. Definition for birth prevalence = (the number of cases with a birth defect in a defined area and time period × 10, 000) ÷ (the number of live births in that area and time period. Definition for point prevalence = (the number of cases of a defect of any age in a defined area on a given date) ÷ (entire population in a defined area on a given date) [24]. Studies which provided the numbers of cases diagnosed with Morquio A were converted to birth prevalence (if there was a time frame and birth statistics for that given time and country). Estimates of point prevalence of Morquio A (and numbers of living patients), were calculated for studies which used recommended diagnostic techniques using the following calculation; birth prevalence × (MPS IVA life expectancy/population life expectancy). Population numbers for countries and region were taken from https://www.cia.gov/library/publications/the-world-factbook/fields/2102.html#138 and Wikipedia respectively. These estimates assumed that the mean life expectancy of Morquio A patients was 25.3 years [3] to account for patients who may have died during the study period. The results were discussed narratively.

## Results

**Figure 1:**
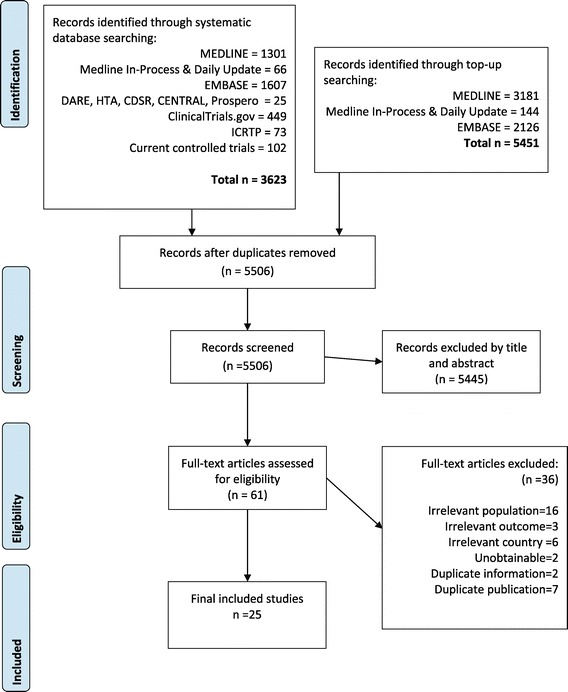
**Flow diagram of included studies.**

In total, 9, 074 records were retrieved from database searches and 25 were included for data extraction. Figure 1 summarises the flow of records through the search and screening process. Of the 61 full records that were screened, 36 did not meet the inclusion criteria and were excluded. Seven were duplicates; the reasons for the remaining 29 exclusions are listed in Additional file 3. Twenty out of 40 key opinion leaders (KOL) and patient organisations (POR) responded to our communications (50%) of which 9 provided data (23%). Two KOL’s provided additional data not included in their publications [25,26]. Analysis of the included data indicated that 4 KOL and 11 published studies provided data for cases or prenatal diagnoses which could not be used to calculate prevalence and were excluded (summarised in Additional file 4). In total we included 5 sets of prevalence data from KOL and PO, and 13 published studies containing prevalence data from database searches. Of the 24 countries of interest, we found data for 13, namely, Australia, Brazil, Canada, Colombia, Denmark, Germany, Japan, Malaysia, Netherlands, Saudi Arabia, Taiwan, UAE, and UK.

### Study characteristics

Study characteristics were summarised in Table 1 and patient characteristics in Additional file 5. All studies were reported to be case series or epidemiological studies of 1 to 34 year duration. The diagnostic methods employed by the different reports were not described in detail. Eight reported the use of enzyme analyses for all patients [27-34] and 6 reported that this was performed in leukocytes or fibroblasts [27-29,32-34]. Two studies reported methods for genotyping [25,34].

**Table 1:** **Characteristics of included studies reporting prevalence data**

**Study**	**Study design**	**Funding source**	**Study duration (years)**	**Study setting/Data source**	**Criteria for diagnosis**	**Methods for genotyping**
KOL Australia [27]	Case series	NR	33	National Referral Laboratory and QLD laboratory Australian Bureau of Statistics	Enzyme assay performed on fibroblasts and/or leukocytes and/or molecular genetic testing	NR
Meikle 1999 Australia [31]	Retrospective case study	Public	16	National Referral Laboratory, Dept. Chemical Pathology, Women’s and Children’s Hospital (Adelaide) and the Division of Chemical Pathology, Royal Brisbane Hospitals (Brisbane).	Enzymatic analyses.	NR
Nelson 2003 Australia (W) [18]	Epidemiological study	NR	27	Genetic and hospital files: Princess Margaret Hospital for Children & King Edward Memorial Hospital for Women, Perth. Medical files: Disability Services Commission of W. Australia, Perth. Laboratory records: Dept. Chemical Pathology, Women’s and Children’s Hospital, Adelaide. The Dept. Clinical Biochemistry, Princess Margaret Hospital for Children, Perth. Membership list of the Society for MPS Diseases (W. Australian Parents Support Group). Records of the Birth Defects Registry of W. Australia.	Diagnosis was confirmed by one dimensional electrophoresis of urinary GAG and/or by enzyme assay on leucocytes or fibroblasts.	NR
KOL Brazil [36]	Retrospective case series	NR	9	Diagnoses recorded by MPS Brazil Network from 2004 until 2014	NR	NR
Applegarth 2000 Canada [30]	Case series	NR	28	Diagnoses made at the Biochemical Diseases Laboratory, Children’s Hospital, Vancouver. Birth records from the British Columbia Vital Statistics Agency.	Specific enzyme assays.	NR
Lowry 1971 Canada (BC) [40]	NR	NR	16	Multiple sources of ascertainment: discharge diagnoses from all inpatients in the hospitals in BC, children’s outpatient’s clinics, public health units, residential institutions for the mentally retarded, the physician’s notice of birth, private physicians and special treatment centres.	NR	NR
Lowry 1990 Canada (BC) [41]	Case series	Public	34	The Biochemical Diseases Laboratory (BDL) of the Department of Pathology, B.C.’s Children’s Hospital, Vancouver and The B.C. Health Surveillance Registry.	NR	NR
Gomez 2012 Colombia (B&C) [39]	Retrospective case series	Public	9	Record histories: Genetics Laboratory The Victoria Hospital, Bogota (Cundinamarca), District Department of Health, El Salvador, of Ubaté (Cundinamarca), Genetics Outpatient Clinic, and patient care area of municipalities (Fúquene, Simijaca Sutatausa, Susa, Carmen de Carupa, Cucunubá, Lenguazaque, Guachetá Tausa, Boyaca. Medical records of the Institute of Human Genetics at the Pontificia Universidad Javeriana, Bogota, and the databases of patient records affiliates to ACOPEL (Colombian Association of Patients with Lysosomsal Disease). Data for live births were obtained from the Dept. National Statistics (DANE).	All clinical assessment was by a specialist in clinical genetics. The diagnosis was confirmed by electrophoresis GAG in urine test determining enzyme levels in leukocytes performed in specialized laboratories or by both techniques widely used in practice clinical diagnosis of mucopolysaccharidosis.	NR
Malm 2008 Denmark [33]	Retrospective case series	NR	30	In Denmark; until 2003: the Kennedy Institute in Glostrup and the Dept. Clinical Genetics at Rigshospitalet, Copenhagen. Today all diagnostics is performed at Rigshospitalet.	Analysis of GAGs in urine (heparan-, dermatan- and keratansulphate) in persons with a clinically suspected MPS. When urinary levels of GAGs are increased, the findings of a low or deficient enzyme level in lymphocytes or fibroblasts confirm the diagnosis of MPS I–MPS VII	NR
Baehner 2005 Germany [32]	Retrospective epidemiological study	NR	15	The identification of affected patients was attained using the following ascertainment sources: 1. Membership list of the German Society for MPS. 2. Patient records from (a) Children’s Hospital, University of Mainz (b) Dept. Pediatrics, and University of Hamburg. 3. Laboratory records from (a) University of Gottingen, (b) University of Munster, (c) University of Heidelberg, (d) University of Greifswald, (e) University of Mainz.	In all cases the diagnosis was confirmed by enzyme assay in serum, leukocytes and/or fibroblasts.	NR
POR Japan [35]	NR	NR	20	Japanese MPS Society	The diagnoses have been performed generally based on enzyme analysis.	NR
KOL Malaysia [28]	Retrospective case series	NR	1	Data collected from 4 major hospitals in Malaysia with clinical genetics service (Hospital Kuala Lumpur, Hospital Pulau Pinang, Hospital Universiti Sains Malaysia, University Malaya Medical Centre).	Reduced GALNS enzyme activity in leukocytes	NR
Poorthuis 1999 Netherlands [37]	Case series	NR	26	The records from the laboratories of the clinical genetic centres involved in the post- and prenatal diagnosis of LSD. The main referral laboratories for the diagnosis of LSD, viz. the clinical genetics centres of Leiden, Nijmegen and Rotterdam, contributed 95% of all cases. Additional information was obtained from the other contributing laboratories.	Cases were enzymatically confirmed, but no details were given.	NR
Moammar 2010 Saudi Arabia (EP) [34]	Retrospective case series	NR	25	Main medical centre in Dhahran, Eastern Province of Saudi Arabia. Birth Statistics from Mortality and Morbidity Reports 1983–2008, Epidemiology Services Unit, Preventive Medicine Services Division, Saudi Aramco Medical Services Organization (SAMSO).	Diagnosis of all glycogen and lysosomal storage disorders was confirmed by enzyme activity estimation on cultured skin fibroblasts, liver biopsy, or leukocytes.	PCR of mithochondrial DNA, oligonucleotide probes to evaluate deletions/rearrangements.
Lin 2009 Taiwan [38]	Retrospective case series	Public	21	Data obtained from: (1) Membership list of Taiwan MPS Society (2) Medical records from (a) Mackay Memorial Hospital, Taipei, Taiwan (b) Taipei Veterans General Hospital, Taipei, Taiwan (c) China Medical University Hospital, Taichung, Taiwan (d) Kaohsiung Medical University Chung-Ho Memorial Hospital, Kaohsiung, Taiwan (e) Kaohsiung Veterans General Hospital, Kaohsiung, Taiwan (f) National Cheng Kung University Hospital, Tainan, Taiwan (g) Buddhist Tzu Chi General Hospital, Hualien, Taiwan (h) Chang Gung Children’s Hospital, Taoyuan, Taiwan (i) Tri-Service General Hospital, Taipei, Taiwan (j) National Taiwan University Hospital, Taipei, Taiwan (3) Laboratory records from Department of Medical Research, Mackay Memorial Hospital, Taipei, Taiwan (4) Records of Taiwan Foundation for Rare Disorders (5) Records of Bureau of Health Promotion, Department of Health, R.O.C. (Taiwan).	The diagnosis of all patients was confirmed by two-dimensional electrophoresis of urinary GAGs and/or enzyme assay in serum, leukocytes and/or fibroblasts.	NR
Al-Jasmi 2010 UAE [25]	Case series	NR	15	Two metabolic referral centres in UAE, Latifa Hospital (Dubai), Tawam Hospital (Abu Dhabi).	Clinical presentation and biochemical analysis.	Direct genomic sequencing of GALNS gene for MPS IVA.
Nelson 1997 UK (NI) [17]	Case series	NR	27	These were hospital consultants’ records; records of the screening laboratory for urinary mucopolysaccharides; the diagnostic indices of the Dept. Medical Genetics; The Queens University of Belfast, The Royal Victoria Hospital, Belfast and The Royal Belfast Hospital for Sick Children; and files of the Hospital activity analysis (Nelson 1986).	Urinary GAGs were examined by 2D electrophoresis. In all cases where the patient was alive at the time of the study, the diagnosis was confirmed by he appropriate enzyme assay.	NR
KOL UK [29]	Case series	NR	38	Whole of the UK including devolved nations Wales, Scotland and Northern Ireland. Data provided by KOL.	Diagnosis will have been made by laboratory on urine GAGS and enzyme analysis. Some will also have mutational analysis.	NR

Abbreviations: *BC* British Columbia, *B&C* Boyacá and Cundinamarca, *DNA* Deoxyribonucleic acid, *EP* Eastern Province, *GAG* Glycosaminoglycan, *GALNS* N-acetylgalactosamine-6 sulfatase, *IEM* Inborn errors of metabolism, *KOL* Key opinion leader, *LSD* Lysosomal storage disorder, *MPS* Mucopolysaccharidoses, *NI* Northen Ireland, *NR* Not recorded, *PCR* Polymerase chain reaction, *POR* Patient organisation representative, *UAE* United Arab Emirates, *UK* United Kingdom.

None of the included studies from literature searching specifically aimed to investigate Morquio A, but were concerned with investigating prevalence in more than one disease e.g. MPSs or LSDs. The majority of studies reported data for Morquio A and Morquio B [17,18,28,29,31,32,35-38], 4 studies reported unspecified Morquio [33,34,39,40] and 4 studies reported data for Morquio A only [25,27,31,41]. Few studies reported details of patient characteristics: 4 studies reported gender [27-29,36], 5 reported age [29,31,33,35,36], 1 reported genotype [25]; 7 reported ethnicity or geographical background [25,27,29,32,33,36,39] and 2 studies reported consanguinity [25,34].

### Quality of study reporting

To assess the quality of reporting of the published studies we adapted the STROBE guidelines by selecting recommendations most relevant to rare diseases (see Additional file 2) [23]. The quality of reporting was summarised in Table 2. None of the studies were judged to have a high quality of reporting; 7 studies were judged as medium [18,25,30-32,37,39], and 6 were judged to be low [17,33,34,38,40,41]. No studies (0%) provided adequate descriptions of the included participants; 10/13 (77%) studies provided adequate descriptions of study design; 3/13 (23%) studies adequately described the eligibility criteria of the participants; 11/13 (85%) studies provided sufficient information to determine that the study population was representative of the target population and 7/13 (54%) studies adequately described the outcomes.

**Table 2:** **Quality of reporting of included studies**

**First author and publication year**	**Country**	**1. ** **Was there an adequate description of study design?**	**2. Was there an adequate description of eligibility criteria?**	**3. Is the study population representative of the target population?**	**4. Is there an adequate description of outcomes?**	**5. Is there an adequate description of the study participants?**	**Overall assessment**
Meikle 1999 [31]	Australia	Yes	Yes	Yes	No	No	Medium
Lowry 1990 [41]	Canada (BC)	No	Unclear	Yes	No	No	Low
Lowry 1971 [40]	Canada (BC)	No	Unclear	Unclear	No	No	Low
Applegarth 2000 [30]	Canada (BC)	Yes	Yes	Yes	Yes	No	Medium
Nelson 2003 [18]	Australia (W)	Yes	No	Yes	Yes	No	Medium
Gomez 2012 [39]	Colombia (B&C)	Yes	No	Yes	Yes	No	Medium
Malm 2008 [33]	Denmark	No	Unclear	Yes	Yes	No	Low
Baehner 2005 [32]	Germany	Yes	Yes	Yes	No	No	Medium
Poorthuis 1999 [37]	Netherlands	Yes	Unclear	Yes	Yes	No	Medium
Moammar 2010 [34]	Saudi Arabia	Yes	Unclear	No	Yes	No	Low
Lin 2009 [38]	Taiwan	Yes	Unclear	Yes	No	No	Low
Al-Jasmi 2010 [25]	UAE	Yes	Unclear	Yes	Yes	No	Medium
Nelson 1997 [17]	UK (NI)	Yes	Unclear	Yes	No	No	Low

Abbreviations: *BC* British Columbia, *B&C* Boyacá and Cundinamarca, *NI* Northern Ireland, *UAE* United Arab Emirates, *UK* United Kingdom.

Quality assessment was based on the checklist in Additional file 2.

Additional file 6 lists all identified study limitations reported by the included studies. Important points raised were lack of central registration system, referral centres or neonatal screening, lack of patient characteristics, movement of patients out of the country of interest during long study periods, lack of study reporting adhering to guidelines, difficult diagnosis, clinical heterogeneity or severity and underdiagnoses due to mild cases and lack of trained staff.

### Summary tables of epidemiology of Morquio A

Only 1 published study and 3 KOL reported point prevalence data (Table 3). Point prevalence of Morquio (unclassified) in Denmark was 1 per 323, 000 as of 31st December 2007 [33]. In Australia, in 2013, point prevalence of Morquio A was 1 in 926,000 [27] while in Malaysia it was 1 per 1,872,000 [28]. In the UK, based on figures from 2011 census, point prevalence for Morquio A was 1 per 599,000 [29]. These results were based on studies with a low or ungraded quality of reporting. In UK and Denmark studies it was reported that the prevalence was influenced by the presence of the disease in Pakistani immigrants.

**Table 3:** **Summary of point prevalence data**

	**MPS IV type**	**Point Prevalence**	**per 10,000**	**Evidence of consanguinity**	**Evidence of ethnicity founder effect**	Representative of whole country**	**Point in time**	**Recommended Enzymatic analysis** ^**ǂ**^	**Quality**
**Australia KOL** [27]	MPS IVA	1 in 926,000*	0.0108*	NR	NR	Y	30th June 2013	Y	ungraded
**Denmark** [33]	MPS IV (unclassified)	1 per 323, 000*	0.031*	NR	Y	Y	31st Dec 2007	Y	low
**Malaysia KOL** [28]	MPS IVA	1 per 1,872,000	0.005*	NR	NR	Y	30th June 2013	Y	ungraded
**UK KOL** [29]	MPS IVA	1 per 599,000*	0.0167*	NR	Y	Y	Mid-2010	Y	ungraded

Abbreviations: *KOL* Key Opinion Leader, *NR* Not recorded, *UK* United Kingdom, *Y* Yes.

*Calculated **An attempt has been made to achieve full ascertainment. ^ǂ^Fibroblast & Leukocyte based analysis.

Birth prevalence was summarised in Table 4 (Morquio A), Table 5 (Morquio unclassified). The results were stratified according to whether or not the recommended diagnostic enzymatic analysis in leukocytes or fibroblasts was used. Other factors which may influence the reliability of the data were also summarised (evidence of consanguinity, evidence of ethnicity founder effect and study period).

**Table 4:** **Summary of birth prevalence for Morquio A**

	**Birth prevalence**	**per 10, 000**	**Evidence of consanguinity**	**Evidence of ethnicity founder effect**	**Representative of whole country**	**Study period**	**Age at diagnoses**	**Cases born during study period** ^**Ɨ**^	**Denominator = live births**	**Recommended enzymatic analysis**	**Quality**
**Australia KOL** [27]	1 per 253,000	0.04*	NR	NR	Y	24 (1980–2013)	0-45	Y	Y	Y^ǂ^	ungraded
**Germany** [32]	1 per 263,000*	0.0380*	NR	Y	Y	15 (1980–1995)	NR	NR	Y	Y^ǂ^	medium
**UAE (Emirates)** [25]	1 per 71, 000	0.14*	Y	NR	N	15 (1995–2010)	NR	Y	Y	Y^ǂ^	medium
**Australia** [31]	1 per 201, 000	0.0497*	NR	NR	Y	17 (1980–1996)	0-19	N^Ɨ^	Y	Y	medium
**Canada (BC)** [30]	1 per 207,000*	0.048*	NR	NR	N	24 (1972–1996)	NR	NR	Y	Y	medium
**Japan (POR)** [35]	1 per 500,000	0.02*	NR	NR	unclear	20 (1991–2011)	NR	NR	Y	Y	ungraded
**Australia (W)** [18]	1 per 641, 000	0.0156*	NR	NR	N	27 (1969–1996)	NR	Y	Y	unclear	medium
**Netherlands** [37]	1 per 459, 000*	0.022*	NR	NR	Y	26 (1970–1996)	NR	Y	Y	unclear	medium
**Taiwan** [38]	1 per 304,000	0.033*	NR	NR	Y	20 (1984–2004)	NR	NR	Y	unclear	low
**UK (NI)** [17]	1 per 76, 000	0.13*	NR	NR	N	27 (1958–1985)	NR	NR	Y	unclear	low
**Brazil (KOL)** [36]	1 per 1,179,000*	0.009*	NR	NR	Y	9 (2004–2013)	1-6	Y	Y	NR	ungraded
**Canada (BC)** [41]	1 per 216, 000	0.046*	NR	NR	N	34 (1952–1986)	NR	NR	Y	NR	low

Abbreviations: *BC* British Columbia, *KOL* Key Opinion Leader, *POR* Patient organisation representative, *N* No, *NI* Northern Ireland, *NR* Not recorded, *UAE* United Arab Emirates, *UK* United Kingdom, *W* West, *Y* Yes. *Calculated by authors; ^Ɨ^assumed from the study period and age at diagnosis; ^ǂ^Fibroblast & Leukocyte based analysis.

**Table 5:** **Summary of birth prevalence for Morquio (unclassified)**

	**Birth prevalence**	**PER 10, 000**	**Evidence of consanguinity**	**Evidence of ethnicity founder effect**	**Representative of whole country**	**Study period**	**Age at diagnoses**	**Cases born during study period** ^Ɨ^	**Denominator = live births**	**Recommended enzymatic analysis**	**Quality**
**Saudi Arabia (Eastern Province)** [34]	1 per 28, 000*	0.362*	Y	N	N	25 (1983–2008)	NR	Y	Y	Y^ǂ^	low
**Denmark** [33]	1 per 208, 000*	0.048*	N	Y	y	29 (1975–2004)	NR	NR	Y	Y^ǂ^	low
**Colombia (B&C)** [39]	1 per 147, 000*	0.068*	N	N	N	9 (1998–2007)	NR	Y	Y	unclear	medium
**Canada (BC)** [40]	1 per 303,000	0.033*	N	N	N	16 (1952–1968)	NR	NR		NR	low

Abbreviations: *BC* British Colombia, *B&C* Boyacá and Cundinamarca, *KOL* Key Opinion Leader, *N* No, *NR* Not recorded, *UK* United Kingdom, *Y* Yes.

*calculated by authors; ^Ɨ^assumed from the study period and age at diagnosis; ^ǂ^Fibroblast & Leukocyte based analysis.

Six studies from Australia, Canada, Germany, Japan and UAE presented data for Morquio A birth prevalence and used the recommended enzymatic analysis. For these studies prevalence ranged from 1 per 71,000 [25] to 1 per 500, 000 [35]. The reliability of these figures was compromised by unclear reporting of whether or not the cases were born during the study and all had long study periods (15-24 yrs). In addition, in Germany it was reported that there was a founder effect from Turkish immigrants (22% of the patients) [32] and in UAE, consanguinity may have influenced the prevalence data as birth prevalence was reported for emirates and not non-emirates (who constitute 80% of the population of UAE) [25]. The Emirate population are described as ethnically diverse and included over 70 distinct tribes which had few inter-tribal marriages. Six studies from Australia, Brazil, Canada, Netherlands, Taiwan and UK (NI) presented data for Morquio A birth prevalence but it was unclear if they had used the recommended enzymatic analysis. For these studies prevalence ranged from 1 per 76,000 [17] to 1 per 1,179,000 [36]. The reliability of these figures was compromised by unclear reporting of whether or not the cases were born during the study [29,38,41] and all except Brazil (9 years) [36] had long study periods (15–34 years). Nelson et al. reported birth prevalence in Northern Ireland, a small and isolated region and therefore arguably not representative of the UK [17]. In addition, the text reports the inclusion of mild cases of Morquio A [17].

**Figure 2:**
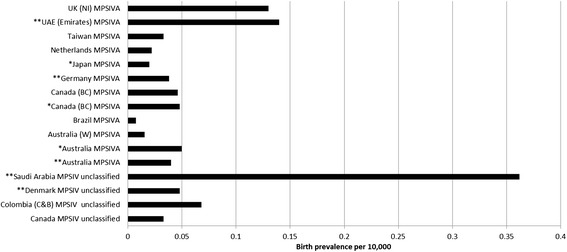
**Birth prevalence of Morquio A and Morquio unclassified.**

Table 5 presents birth prevalence for Morquio (unclassified). Two studies in Denmark and Saudi Arabia used the recommended diagnostic method [33,34]. The prevalence of these countries ranged from 1 per 28, 000 to 1 per 208, 000. The limitations of Denmark were discussed above. The study of Saudi Arabia investigated Morquio (unclassified) specifically in the eastern province and used the medical records from an oil company; therefore it was not representative of the country. In addition, the result for Saudi Arabia was further compromised by evidence of consanguinity (patients were frequently siblings and all had consanguineous parents), poor quality of study reporting and the study was carried out over a long period (25 years) [34]. Two studies from Colombia and Canada presented data for Morquio (unclassified) birth prevalence but it was unclear if they had used the recommended enzymatic analysis [36,38-40]. For these studies prevalence ranged from 1 per 147, 000 to 1 per 303, 000. The reliability of Canada was discussed above. The data for Colombia were not representative of the whole country [39]. All birth prevalence data are illustrated in Figure 2.

### Estimated point prevalance and number of cases alive from reported birth prevalence

To try and overcome the limitations that restricted life span imposes on calculations of prevalence, particularly performed on data collected over many years we tried to estimate point prevalence (and numbers of people alive with MPS Morquio A). Estimations were performed for the studies with the recommended diagnosis techniques only and the results are summarised in Table 6. Estimated point prevalence range from 1 per 1,664,000 in Japan (estimated 76 cases) to 1 per 217,000 in UAE (estimated 25 cases). The estimates are averaged over the study period and are still compromised by the limits discussed above.

**Table 6:** **Estimations of Morquio A point prevalence from birth prevalence data**

	Birth prevalence (per 10, 000)*	**Estimated MPS IVA point prevalence** ^**#**^	**Estimated MPS IVA point prevalence per 10,000**	**Estimated people alive with MPSIVA**
**Australia KOL** [27]	0.04	1 per 810,000	0.0123	27
**Germany** [32]	0.038	1 per 835,000	0.0120	97
**UAE (Emirates)** [25]	0.14	1 per 217,000	0.0460	25
**Australia** [31]	0.0497	1 per 652,000	0.0153	34
**Canada (BC)** [30]	0.048	1 per 668,000	0.0150	7
**Japan (POR)** [35]	0.02	1 per 1,664,000	0.0060	76

Abbreviations: *BC* British Columbia, *KOL* Key Opinion Leader, *POR* Patient organisation representative, *NI* Northern Ireland, *UAE* United Arab Emirates, *UK* United Kingdom, *W* West. *Calculated by authors from Tables 4 and 5; ^**#**^average point prevalence for study period.

Estimated MPS IVA patients alive = MPSIVA point prevalence × population.

## Discussion

Point prevalence was reported by the following countries: Australia (1 per 926,000) [27]; Denmark (unclassified Morquio = 1 per 323, 000) [33]; Malaysia (1 per 1,872,000) [28] and UK (1 per 599, 000) [29]. Birth prevalence was reported by the following countries: Australia, Brazil, Canada, Germany, Japan, Netherlands, Saudi Arabia, Taiwan, UAE and UK. For Morquio A birth prevalence ranged from 1 per 71, 000 [25] to 1 per 500, 000 [35] for studies with recommended diagnostic methods. For all unclassified Morquio, birth prevalence ranged from 1 per 28, 000 [34] to 1 per 208, 000 [33] for studies with recommended diagnostic methods. No data were found for prevalence of Morquio A in France, Italy, Mexico, Poland, Portugal, Qatar, Spain, South Korea, Turkey or United States of America (USA).

All results for prevalence were compromised by poor study reporting and internal validity (flaws in the design or conduct of the study) as detailed in the quality and results sections. Contacting the KOL and POR resulted in a poor response rate; given time more information may have been forthcoming. A systematic review proved an effective method of obtaining information on prevalence of Morquio A. To our knowledge this is the first systematic review of its kind, investigating the prevalence of Morquio A in multiple countries.

### Challenges of performing a systematic review of prevalence of a rare disease

Few systematic reviews exist on the prevalence of rare conditions. For Morquio A, there was limited information on prevalence. When attempting to retrieve studies difficulties may arise if Morquio A is included within a broader report investigating lysosomal storage disorders, metabolic disorders, MPS diseases or inborn errors. In practical terms it can be challenging to identify and screen studies for records of Morquio A or MPS IVA if it is only one of many conditions. In our current research we noted that Morquio A may not be referred to in the title or abstract, but may appear only in a table. Therefore screening on the title and abstract alone for specific diseases would lead to the loss of important documents and therefore it is important to be aware of categories under which rare diseases may be grouped.

Assessment of study quality is an essential part of a systematic review. For this review we adapted the STROBE checklist [23]. As judged by our assessment, no studies were reported well. In general there was a lack of reporting for the method of diagnosis; type of Morquio (i.e. Type A or B) and no studies adequately described the characteristics of study participants. The lack of detail for patient characteristics highlights a loss of valuable information on the potential effects of ethnicity and consanguinity on prevalence. Also, it was generally unclear if the study population was representative of the target population due to the issues surrounding underestimation and overestimation of patient numbers. This is discussed further in study limitations. The STROBE guidelines may not be the most suitable tool for assessing the quality of prevalence studies in rare diseases as they are designed to assess observational studies and often the questions do not apply to such small populations, this is clearly an area for future research. There are no checklists for the assessment of methodological quality of rare disease studies. We have tried to account for this using the fields in Tables 3, 4 and 5 relating to consanguinity, ethnicity, study period, diagnosis and definition of prevalence. These fields allowed us to create a judgement as to whether the reported prevalence is compromised and methodological quality should be explored further in future research.

### Study limitations

Overall the advancement and improvement of research for rare diseases is hindered by lack of funding and lack of samples. We have discussed that there are several areas within the systematic review process that may limit the current study (searching, screening and quality assessment) however there are further limiting issues specific to this study (prevalence definition and diagnosis).

There was a great deal of misunderstanding regarding how prevalence data should be reported. Terms such as incidence, prevalence, frequency and birth prevalence were all used (some included either prenatal or postnatal diagnoses or both) and often where calculations were fully described it could be seen that the terms were used interchangeably. As studies report different types of prevalence we could not easily make comparisons, therefore we grouped together studies which reported similar definitions using point prevalence or birth prevalence (in agreement with the NBDPN guidelines). When analysing birth prevalence studies rarely reported ‘diagnosis at birth’. Classical presentation of Morquio A is usually diagnosed during early childhood; screening programmes for rare diseases are impractical and expensive. In addition since Morquio A is a recessive disorder the parents are unaffected by the disease and so there is no obvious reason to test at birth. However, for the purposes of this review it was important to differentiate between those studies which included cases ‘born during the study period’ and ‘those diagnosed during the study period’ to produce accurate birth prevalence calculations. The vast majority of studies that reported ‘prevalence’ actually reported ‘birth prevalence’. In our review only one study and three KOL reported point prevalence (prevalence at a single point in time) [27-29,33]. We considered point prevalence to be a more accurate measure of prevalence than birth prevalence as it is not influenced by the length of study period. Birth prevalence may not accurately reflect the number of patients alive with the disease, since there is the possibility that patients who are born within a study may also die or migrate from a country during the study period. The longer the period of the study the more chance that either can happen; this effect does not influence point prevalence. Birth prevalence figures from shorter study periods are more likely to reflect patients alive than the longer study periods. The recent paper by Lavery and Hendriksz 2014 indicated that 19% (5/27) of patients with Morquio A in the UK had died before the age of 10 [3]. To overcome these issues we also attempted to estimate point prevalence from birth prevalence (Table 6). These results are an average for the study period and therefore do not provide a true reflection of current Morquio A populations and are still open to all other forms of bias.

Prevalence is clearly very dependent on accurate diagnosis and any review should consider how well this was performed. To compare the prevalence of Morquio A we stratified the results according to whether or not the studies were diagnosed using the recommended enzyme analysis in fibroblasts and leukocytes. Those using the recommended analysis were considered to be of better methodological quality. However, the technique of enzyme analysis is not perfect and can yield false negatives when the enzyme is present at low quantities [10]. Low levels of enzyme activity can also be found if the sample has degraded during storage and therefore storage of samples is an important point to consider especially in studies performed over a long time period, consideration of this is important as it may lead to an underestimation of prevalence.

Underestimation of prevalence occurs if not all patients were included in the study and this may be due to late diagnosis, mild forms of the disease and lack of screening. Potential exists for over-estimation of prevalence. In studies with multiple sources of ascertainment of data, it is possible, for a patient to be diagnosed at a hospital and to exist as a member of a patient support group. This may result in double counting of patients. We have attempted to avoid double counting when we retrieved multiple references for a given country but it is difficult without detailed patient characteristics. No studies reported any additional enzymatic testing to rule out diseases such as MSD, MLII or MLIII as recommended by Wood et al. 2013, so there is potential for mis-diagnosis and over-estimation of Morquio A patient numbers. Similarly, populations where consanguinity is typical and migration of ethnic groups where Morquio A is more common will also result in above average prevalence of Morquio A. Often no distinction was made between the sub-types. For some studies this may reflect the use of data from pre-1976 (when the two diseases were first identified) or it may also indicate the lack of available accurate diagnostics test during a time period or for a given region or lack of disease awareness.

Given the limitations discussed surrounding the reporting of prevalence data in this field we have made recommendations to aid future research and hopefully produce more accurate and comparable prevalence data on rare diseases. Some recommendations may be already be supported by European Project for Rare Diseases National Plans Development (EUROPLAN) [42].

### Recommendations for systematic reviewers

- Development of a new set of reporting guidelines and a quality assessment tool specific to rare disease prevalence studies with specific emphasis on 1) definition of disease, 2) method of diagnosis and sample storage, 3) age at time of diagnosis, 4) ascertainment of data, 5) definition of prevalence, and 6) suitability of measurement.

### Recommendations for clinicians

- Use birth prevalence as defined by NBDPN guidelines or point prevalence to record occurrence of Morquio A in populations. Do not use prenatal diagnoses unless live births result.
- A consensus should be reached on how to report prevalence of rare diseases such as Morquio A considering the challenges faced. With Morquio A in particular, important points are 1) enzymatic diagnosis, 2) defining and reporting type A and B separately, 3) genetic analysis of patients, 4) clear definition of point and/or prevalence 5) reporting patient characteristics. This would make comparisons possible across countries and provide a more accurate assessment of the true prevalence of Morquio A and similar conditions.
- An obligatory reporting or national registration scheme should be set up for each country in order to collate information on patients and provide access to treatment.

## Conclusions

Morquio A point prevalence was found to be 1 per 926,000, 1 per 599, 000; 1 per 1,872,000 in Australia, UK and Malaysia respectively. In Denmark, point prevalence of unclassified Morquio was 1 per 323, 000. Birth prevalence for Morquio A ranged from 1 per 71,000 to 1, 179,000 across multiple countries. All results were compromised by poor study reporting and internal validity. It is important to have an accurate reflection of the prevalence of Morquio A in order to efficiently direct treatment, funds and resources. Evidence of both over-representation and under-representation of Morquio A exists. Bespoke reporting guidelines, a quality assessment tool specifically for prevalence of rare diseases and agreed best diagnostic methods or screening programmes would be a practical way to help clinicians produce more meaningful data and allow comparison across populations.

## Additional files

Additional file 1:
**Search strategies.**
Additional file 2:
**Checklist for reporting items in observational studies of rare diseases (adapted from strobe checklist).**
Additional file 3:
**Excluded studies.**
Additional file 4:
**Non-comparable studies.**
Additional file 5:
**Patient characteristics.**
Additional file 6:
**Reported study limits.**
