# Supplementary material for: A systematic review of the prevalence of Morquio A syndrome: challenges for study reporting in rare diseases
Source: Orphanet J Rare Dis. 2014 Nov 18;9:173. doi: 10.1186/s13023-014-0173-x (PMC4251694; doi:10.1186/s13023-014-0173-x)
Supplement: Additional file 1: — Search strategies. [file 13023_2014_173_MOESM1_ESM.docx]

**Additional file 1: Search Strategies**

**Embase (OvidSP): 1974-2013/09/13**

**Searched 16.9.13**

1 Morquio syndrome/ (1117)

2 (morquio$ adj3 (syndrome$ or disorder$ or disease$)).ti,ab,ot,hw. (1291)

3 (Morquio adj2 Brailsford).ti,ab,ot. (57)

4 MorquioA.ti,ab,ot. (0)

5 Morquio$.ti,ab,ot. (798)

6 ((mucopolysaccharidosis or MPS) adj5 (IV or "4" or IVA or IV-A or "4A" or "4-A")).ti,ab,ot. (607)

7 (MPS4A or MPSIVA or MPS4-A or MPSIV-A or MPS4 or MPSIV).ti,ab,ot. (12)

8 (galactose 6-sulfate sulfatase deficien$ adj3 (syndrome$ or disorder$ or disease$)).ti,ab,ot,hw. (0)

9 ((GALN or GALNS) adj3 deficien$).ti,ab,ot,hw. (17)

10 (N-acetylgalactosamine-6-sulfatase adj3 deficien$).ti,ab,ot,hw. (15)

11 familial osseous dystroph$.ti,ab,ot,hw. (2)

12 (kerato sulfaturia$ or keratosulfaturia$).ti,ab,ot,hw. (4)

13 (osteochondrodystrophia deformans or osteo-chondrodystrophia deformans).ti,ab,ot,hw. (3)

14 or/1-13 (1706)

15 animal/ or animal experiment/ (3586833)

16 (rat or rats or mouse or mice or murine or rodent or rodents or hamster or hamsters or pig or pigs or porcine or rabbit or rabbits or animal or animals or dogs or dog or cats or cow or bovine or sheep or ovine or monkey or monkeys).mp. (5814221)

17 or/15-16 (5814221)

18 exp human/ or human experiment/ (14950320)

19 17 not (17 and 18) (4633611)

**20 14 not 19 (1607)**

**Embase (OvidSP): 2000-2013/10/4**

**Searched 7.10.13**

1 exp mucopolysaccharidosis/ep (110)

2 lysosome storage disease/ep (69)

3 1 or 2 (166)

4 (lysosom$ adj2 storage adj2 (disease$ or disorder$ or syndrom$ or defect$ or deficien$)).ti,ab. (5117)

5 (lysosom$ adj2 (disease$ or disorder$ or syndrom$ or defect$ or deficien$)).ti,ab. (6586)

6 (mucopolysacchar$ adj3 (disease$ or disorder$ or syndrome$ or defect$ or deficien$)).ti,ab. (1257)

7 mucopolysaccharidos$.ti,ab. (4090)

8 or/4-7 (9838)

9 incidence/ (209738)

10 prevalence/ (352528)

11 demography/ (137247)

12 exp epidemiology/ or epidemiological data/ (1939489)

13 (frequency or frequencies or frequent$ or occurrence$ or incidence$ or prevalence$ or number$ or prevalent$ or times$ or rate or rates or episode$ or epidemiolo$ or distributed or distributions$ or natural histor$ or demograph$ or survey$).ti,ab,ot,hw. (7494693)

14 or/9-13 (7935296)

15 8 and 14 (3111)

**16 3 or 15 (3181)**
