# Supplementary material for: A systematic review of the prevalence of Morquio A syndrome: challenges for study reporting in rare diseases
Source: Orphanet J Rare Dis. 2014 Nov 18;9:173. doi: 10.1186/s13023-014-0173-x (PMC4251694; doi:10.1186/s13023-014-0173-x)
Supplement: Additional file 2: — Checklist for reporting items in observational studies of rare diseases (adapted from strobe checklist). [file 13023_2014_173_MOESM2_ESM.docx]

**Additional file 2: CHECKLIST FOR REPORTING ITEMS IN OBSERVATIONAL STUDIES OF RARE DISEASES (ADAPTED FROM STROBE CHECKLIST)**

1. **Was there an adequate description of study design and setting? Y, N or UNCLEAR**

**Select YES if authors**:

- described the method of data collection (e.g. retrospective epidemiological survey, records from a list of sources)
- described the setting (e.g. clinics, population registered at general practices, medical records database)
- relevant dates (periods for recruitment, data collection).
- give the source of denominator population for prevalence calculations (e.g. UK national statistics)

**Select NO if authors** did not report all of the above

**Select UNCLEAR** if authors reported design and setting information but it was presented unclearly or incompletely (e.g. the number of general practices was not reported or only the recruitment start date was reported)

1. **Was there an adequate description of eligibility criteria? Y, N or UNCLEAR**

**Select YES if authors:**

- described inclusion criteria (exclusion criteria are not necessary)
- explicitly stated which type of MPS IV they report (specifically MPS IVA or MPS IVB)
- include enzymatic or genetic analysis as diagnoses methods (it is sufficient to state patients were enzymatically diagnosed without giving full details)

**Select NO if authors** did not report all of the above

**Select UNCLEAR** if authors

- reported eligibility criteria but it was presented unclearly (if MPS IVA is reported but no diagnostic method)
- did not clearly state which type of MPS IV was reported

1. **Is the study population representative of the target population? Y, N, UNCLEAR**

Note – for this question, the target population is the population studied in the study, not the population that we are studying for this systematic review. Ethnicity is not important, as long as the patient lives in the given country.

**Select YES if authors:**

State the sources include all necessary diagnostic centres or that they have attempted to achieve full ascertainment or have outlined an extensive list of sources

**Select NO** if there is reason to believe that full ascertainment has not been achieved

**Select UNCLEAR** if we cannot be sure that all patients were included in the study (e.g. in a country multiple centres could have performed the diagnostic analyses and not all participated in the study).

1. **Is there an adequate description of outcomes? Y, N, UNCLEAR**

**Select YES if authors clearly describe:**

- patients in denominator – live births or general population
- patients in numerator were born during study period (birth prevalence) or were living during study period (period prevalence)
- time frame of study
- the period of study eg ‘date of first diagnosed’ case to last diagnosed case or ‘date of birth of first diagnosed case’ to last diagnosed case

**Select NO if authors** did not report all of the above

**Select UNCLEAR** if any of the above are not clearly reported

1. **Is there an adequate description of the study participants? Y, N, UNCLEAR**

**Select YES** if the authors provided more than just age (at diagnosis) and gender (for example ethnicity) then I would say the participants were adequately described

**Select NO if authors** did not report more than age and gender

Select UNCLEAR if the population descriptions were unclear (e.g. numbers in texts and figures didn’t match or add up).

**Overall score:**

High – all criteria met (5 Yes’s)

Medium – 1 to 2 criteria not met (i.e. 1-2 No’s or Unclear)

Low – 3 or more criteria not met (i.e. ≥ 3 No’s or Unclear)
