# Supplementary material for: A systematic review of the prevalence of Morquio A syndrome: challenges for study reporting in rare diseases
Source: Orphanet J Rare Dis. 2014 Nov 18;9:173. doi: 10.1186/s13023-014-0173-x (PMC4251694; doi:10.1186/s13023-014-0173-x)
Supplement: Additional file 3: — Excluded studies. [file 13023_2014_173_MOESM3_ESM.docx]

**Additional file 3: Details of excluded studies**

| **First author and publication year** | **Country** | **Title of Article/Journal** | **Reason for exclude** |
| --- | --- | --- | --- |
| Adams 1982 | USA | Diseases diagnosed by the Shriver Centre Lysosomal Storage Diseases Laboratory during 2-year period. In: Neurology of Hereditary Metabolic Diseases of Children, McGraw-Hill, New York, 1982, p. 299 | Unobtainable |
| Bach 2005 | Israel | The frequency of mucolipidosis type IV in the Ashkenazi Jewish population and the identification of 3 novel MCOLN1 mutations. *Hum Mutat* 2005;26(6):591. | Not relevant population |
| Badalian 1971 | Russia | Genetically determined mucopolysaccharidoses in children. *Pediatriia* 1971;50(3):58-62. | Not relevant population |
| Beighton 1973 | South Africa | Atlanto axial subluxation in the Morquio syndrome: Report of a case. *Journal of Bone and Joint Surgery - Series B* 1973;55(3):478-481. | Not relevant country |
| Beighton 1973 | South Africa | Atlanto-axial subluxation in the Morquio syndrome. Report of a case. *The Journal of bone and joint surgery* 1973;British volume. 55(3):478-481. | Not relevant country |
| Bodamer 2010 | Austria | Newborn screening for lysosomal storage diseases. *Int J Clin Pharmacol Ther* 2010;48(SUPPL. 1):S18-S19. | Not relevant outcome (no prevalence data or number of cases) |
| Cherin 2010 | France | Result of the survey on orphan disorders by the internal medicine--lysosomal diseases group. *Rev Med Interne* 2010;31(7):515-6. | Not relevant population |
| Deodato 2004 | Italy | Deodato F, Boenzi S, Rizzo C, Abeni D, Caviglia S, Picca S, et al. Inborn errors of metabolism: An update on epidemiology and on neonatal-onset hyperammonemia. *Acta Paediatrica, International Journal of Paediatrics, Supplement* 2004;93(445):18-21. | Not relevant population |
| Dionisi-Vici 2002 | Italy | Inborn errors of metabolism in the Italian pediatric population: a national retrospective survey. *J Pediatr* 2002;140(3):321-7. | Not relevant population |
| Fang-Kircher 1995 | Austria | Morquio disease in a patient diagnosed as having Perthes disease for 38 years. *J Inherit Metab Dis* 1995;18(1):94-95. | Not relevant country |
| Galjaard 1980 | Netherlands | Genetic Metabolic Diseases: Early Diagnosis and Prenatal Analysis, Elsevier/North Holland Biomedical Press, Amsterdam, 1980b, pp 93101; 109-112; 266-28t; 290-296 | Unobtainable |
| Giugliani 2012 | Latin America | Newborn screening for lysosomal diseases: current status and potential interface with population medical genetics in Latin America. *J Inherit Metab Dis* 2012;35(5):871-7. | Duplicate information  (reports data from already included publications) |
| Guia 2000 | Spain | Known multiple malformation syndromes and cardiac defects. Study on 1,216 children from Murcia, a region of Spain, during the period of 1978-1990. [Spanish]. *Revista Espanola de Pediatria* 2000;56(334):314-320. | Not relevant population |
| Hutchesson 1998 | UK | A comparison of disease and gene frequencies of inborn errors of metabolism among different ethnic groups in the West Midlands, UK. *J Med Genet* 1998;35(5):366-70. | Not relevant population |
| Krasnopolskaia 1992 | Russia | A program of prevention of hereditary lysosomal diseases in the USSR. *Vestn Ross Akad Med Nauk* 1992(4):20-4. | Duplicate information  (reports data from already included publications) |
| Mayer 2009 | Brazil | New GLB1 mutation in siblings with Morquio type B disease presenting with mental regression. *Mol Genet Metab* 2009;96(3):148. | Not relevant outcome (prevalence data or number of cases) |
| Mena 1976 | Chile | Morquio's syndrome. [Spanish]. *Rev Chil Pediatr* 1976;47(3):247-254. | Not relevant country |
| Montano 2007 | Multiple | International Morquio A Registry: Clinical manifestation and natural course of Morquio A disease. *J Inherit Metab Dis* 2007;30(2):165-174. | Not relevant population (not newly diagnosed cases) |
| National MPS Society | USA | National MPS Society, Study to Detect Unrecognized Mucopolysaccharidosis in Children Visiting Rheumatology, Hand or Skeletal Dysplasia Clinics. <http://ClinicalTrials.gov/show/NCT01675674> Last updated: May 23, 2013 | Not relevant outcome (no results published) |
| Palandurkar 2011 | India | The diagnosis of Morquio disease correlating the clinical, radiological and biochemical findings: A case series. *Journal of Clinical and Diagnostic Research* 2011;5(8):1641-1645. | Not relevant country |
| Roche 1992 | Ireland | Chondro-osteodystrophy (Morquio Brailsford disease, mucopolysaccharidosis type IV A). *Ir Med J* 1992;85(4):153. | Not relevant country |
| Sanderson 2006 | UK | The incidence of inherited metabolic disorders in the West Midlands, UK. *Arch Dis Child* 2006;91(11):896-9. | Not relevant population |
| Sanjurjo 2008 | Spain | Inborn errors of metabolism as rare diseases with a specific global situation. *An Sist Sanit Navar* 2008;31 Suppl 2:55-73. | Not relevant population |
| Schwartz 2009 | Brazil | MPS-Brazil network (2004 - 2009): Epidemiological update on mucopolysaccharidosis in Brazil. In: Molecular Genetics and Metabolism. Conference: 11th International Congress of Inborn Errors of Metabolism San Diego, CA United States. Conference Start: 20090829 Conference End: 20090902. Conference Publication: (var.pagings). 98 (1-2) (pp 77), 2009. Date of Publication: September-October 2009., 2009. | Not relevant population |
| Suzuki 2009 | Japan | Mucopolysaccharidosis type IV in Japan: A questionnaire survey. In: Molecular Genetics and Metabolism. Conference: 11th International Congress of Inborn Errors of Metabolism San Diego, CA United States. Conference Start: 20090829 Conference End: 20090902. Conference Publication: (var.pagings). 98 (1-2) (pp 87), 2009. Date of Publication: September-October 2009., 2009. | Not relevant population (not newly diagnosed cases) |
| Tylki-Szymanska 2001 | Poland | The prevalence and diagnosis of lysosomal storage diseases in Poland. *Eur J Pediatr* 2001;160(4):261-2. | Not relevant population |
| Vaca 1981 | Mexico | Detection of inborn errors of metabolism in 1,117 patients studied because of suspected inherited disease. *Arch Invest Med (Mex)* 1981;12(3):341-8. | Not relevant population |
| Volkov 1977 | Russia | Morquio's syndrome; mucopolysaccharidosis type IV (clinical and genealogic study). [Russian]. *Ortopediya Travmatologiya i Protezirovanie* 1977;no.7:1-8. | Not relevant population |
| Yamaguchi 2008 | Japan | Newborn screening in Japan: restructuring for the new era. *Ann Acad Med Singapore* 2008;37(12 Suppl):13-5. | Not relevant population |
