# Supplementary material for: A systematic review of the prevalence of Morquio A syndrome: challenges for study reporting in rare diseases
Source: Orphanet J Rare Dis. 2014 Nov 18;9:173. doi: 10.1186/s13023-014-0173-x (PMC4251694; doi:10.1186/s13023-014-0173-x)
Supplement: Additional file 4: — Non-comparable studies. [file 13023_2014_173_MOESM4_ESM.docx]

**Additional file 4: NON-COMPARABLE studies**

| **First author and publication year** | **Country** | **Title** | **Type of MPS IV** | **Reported Birth Prevalence** | **Reported Incidence** | **Reported Cases** | **Reason why not comparable** |
| --- | --- | --- | --- | --- | --- | --- | --- |
| Biomarin Representative | Italy | MPS IV prevalence (Italy) | MPS IVA | NR | NR | 40-50 | Insufficient information to calculate prevalence |
| Biomarin Representative | Malaysia | MPS IV prevalence (Malaysia) | MPS IVA | NR | NR | 14 | Insufficient information to calculate prevalence |
| Biomarin Representative | South Korea | MPS IV prevalence (South Korea) | MPS IVA | NR | NR | 13 | Insufficient information to calculate prevalence |
| Coelho 1997 | Brazil & Latin America | R. Selective screening of 10,000 high-risk Brazilian patients for the detection of inborn errors of metabolism. Eur J Pediatr. 1997 Aug;156(8):650-4. | MPS IVA  MPS IVB  MPS IV (unclassified)  MPS IV (total) | NR | NR | 20  3  2  25 | Insufficient information to calculate prevalence |
| Emre 2002 | Turkey | Biochemical and molecular analysis of mucopolysaccharidoses in Turkey. Turk J Pediatr. 2002 Jan-Mar;44(1):13-7. | MPS IVA | NR | NR | 11 | Insufficient information to calculate prevalence |
| Fuentes-Fuentes 2012 | Mexico | [Frequency of mucopolysaccharidoses diseases at the Hospital Infantil de Mexico Federico Gomez]. Rev Invest Clin. 2012 Sep-Oct;64(5):495-6. | MPS IV (unclassified) | NR | NR | 4*  2**  0*** | Insufficient information to calculate prevalence |
| Key Opinion leader | Portugal | MPS IV prevalence (Portugal) | MPS IVA  MPS IVB | NR | NR | 15  2 | Insufficient information to calculate prevalence |
| Krasnopolskaya 1993 | Russia  Former USSR & Central Asia Republics | Diagnosis and prevention of lysosomal storage diseases in Russia. J Inherit Metab Dis. 1993;16(6):994-1002. | MPS IVA  MPS IVB | NR | NR | 23 cases  16 families  3 cases  2 families | Insufficient information to calculate prevalence |
| Krasnopolskaya 1993 | Russia  Central Asia Republics | Diagnosis and prevention of lysosomal storage diseases in Russia. J Inherit Metab Dis. 1993;16(6):994-1002. | MPS IVA  MPS IVB | NR | NR | 11 cases  5 families  0 cases  0 families | Insufficient information to calculate prevalence |
| Krasnopolskaya 1993 | Russia  Former USSR | Diagnosis and prevention of lysosomal storage diseases in Russia. J Inherit Metab Dis. 1993;16(6):994-1002. | MPS IVA  MPS IVB | NR | NR | 12 cases  11 families  3 cases  2 families | Insufficient information to calculate prevalence |
| Krasnopolskaya 1997 | Russia | Postnatal and prenatal diagnosis of lysosomal storage diseases in the former Soviet Union. Wien Klin Wochenschr. 1997;109(3):74-80. | MPS IVA  MPS IVB | NR | NR | 28  3 | Insufficient information to calculate prevalence |
| Ozand 1990 | Saudi Arabia | Prevalence of different types of lysosomal storage diseases in Saudi Arabia. J Inherit Metab Dis. 1990;13(6):849-61. | MPS IVA | NR | NR | 19 | Insufficient information to calculate prevalence |
| Pinto 2004 | Portugal N)  Portugal  Portugal (Other) | Prevalence of lysosomal storage diseases in Portugal. Eur J Hum Genet. 2004;12(2):87-92. | MPS IVA | 0.6 (0.6 per 100 000) | NR | 6  10  5 | Pregnancy outcomes of prenatal diagnoses not reported. |
| Piraud 1993 | France | Diagnosis of mucopolysaccharidoses in a clinically selected population by urinary glycosaminoglycan analysis: a study of 2,000 urine samples. Clin Chim Acta. 1993 Nov 30;221(1-2):171-81. | MPS IVA  MPS IVB | NR | NR | 24  9 | Insufficient information to calculate prevalence |
| Rezende 2012 | Brazil | Diagnosis of mucopolysaccharidosis of a reference center laboratory for inborn errors of metabolism (LEIM): a Brazilian experience. Mol Genet Metab. 2012;105(2):S54. | MPS IV (unclassified) | NR | NR | 16 | Insufficient information to calculate prevalence |
| Uribe 2013 | Colombia | Selective screening for lysosomal storage diseases with dried blood spots collected on filter paper in 4,700 high-risk colombian subjects. JIMD Rep. [Journal Article]. 2013;11:107-16. | MPS IVB | NR | NR | 1 | Insufficient information to calculate prevalence |
| Valadares 2011 | Brazil (Minas Gerais) | Mucopolysaccharidoses in the state of Minas Gerais, Brazil. J Inherit Metab Dis. 2011;34:S207. | MPS IV (unclassified) | NR | NR | 2 | Insufficient information to calculate prevalence |
| Zetina 1989 | Mexico | [Hereditary lysosomal diseases. I. Initial results of a diagnostic program in Mexico]. Rev Invest Clin. 1989;41(4):319-26. | MPS IV (unclassified) | NR | NR | 13 | Insufficient information to calculate prevalence |

*Registry 1 (1943-1975),**Registry 2 (1994-2005)***Registry 3 (2006-2010)
