# Supplementary material for: A systematic review of the prevalence of Morquio A syndrome: challenges for study reporting in rare diseases
Source: Orphanet J Rare Dis. 2014 Nov 18;9:173. doi: 10.1186/s13023-014-0173-x (PMC4251694; doi:10.1186/s13023-014-0173-x)
Supplement: Additional file 5: — Patient characteristics. [file 13023_2014_173_MOESM5_ESM.docx]

**Additional file 5: PATIENT CHARACTERISTICS**

| **Study** | **Reported name for MPSIV** | **Male n (%)** | **Median age**  **(range)** | **Height (m)**  **Weight (kg)** | **Genotype** | **Ethnicity (%)** | **Geographical background** | **Consanguinity** | **Other comments** |
| --- | --- | --- | --- | --- | --- | --- | --- | --- | --- |
| KOL Australia[^1^](#_ENREF_1) | Morquio A  Morquio B | 9 (64) | NR | NR | NR | All but two were Caucasian, with the other 2 of South-East Asian origin. | NR | NR | Patient characteristics only available on 14 patients diagnosed since 1996:  8 were diagnosed at under 3 years of age, 3 more at 3-6 years of age (79% diagnosed <6 yrs), with the remaining 3 at 10, 33 and 45 years of age |
| Meikle 1999  Australia[^2^](#_ENREF_2) | MPS IVA  MPS IVB | NR | 3.4  (0-19)  at diagnosis | NR | NR | NR | NR | NR | If the age at diagnosis = 0, then diagnosis was within the first 2 weeks of birth. |
| Nelson 2003  Australia (W)[^3^](#_ENREF_3) | MPS IVA  MPS IVB | NR | NR | NR | NR | NR | NR | NR | NR |
| KOL Brazil[^4^](#_ENREF_4) | MPS IVA  MPS IVB | 26 | 8  (3-10) | NR | NR | NR | NR (born in Brazil) | NR | NR |
| Applegarth 2000  Canada (BC)[^5^](#_ENREF_5) | Morquio A | NR | NR | NR | NR | Predominantly caucasian. | NR (Born in British Columbia). | NR | NR |
| Lowry 1971  Canada (BC)[^6^](#_ENREF_6) | Morquio syndrome | NR | NR | NR | NR | NR | NR | NR | NR |
| Lowry 1990  Canada (BC)[^7^](#_ENREF_7) | MPS IVA | NR | NR | NR | NR | NR | NR | NR | NR |
| Gomez 2012  Colombia (B&C)[^8^](#_ENREF_8) | MPS IV | NR | NR | NR | NR | NR | 9 cases from Bogotá, 1 from Madrid, 1 from Nemocon and 1 from Tausa, Columbia. | NR | Spatial clustering analysis found that 2 cases of MPS IV clustered in the area Tausa, Ubaté and Nemocon (0.14 expected cases), p = 0.019. |
| Malm 2008  Denmark[^9^](#_ENREF_9) | MPS IV | NR | NR  (1-67) | NR | NR | Ethnicity founder effect in Pakistani population | NR | NR | NR |
| Baehner 2005  Germany[^10^](#_ENREF_10) | MPS IVA  MPS IVB | NR | NR | NR | NR | For Morquio disease type A (MPS IVA), 11 of 51 patients (22%) were of Turkish origin. | There is a large Turkish population in Germany | NR | The number of patients from other ethnic groups was negligible. |
| POR Japan[^11^](#_ENREF_11) | MPS IVA  MPSIVB | 80 | 30.7  (12 - 50) | NR | NR | NR | NR (live in Japan) | NR | NR |
| KOL Malaysia[^12^](#_ENREF_12) | MPS IVA  MPSIVB | NR | NR | NR | NR | NR | NR | NR | NR |
| Poorthuis 1999  Netherlands[^13^](#_ENREF_13) | MPS IV  MPS IVA  MPS IVB | NR | NR | NR | NR | NR | NR | NR | NR |
| Moammar 2010  Saudi Arabia (EP)[^14^](#_ENREF_14) | MPS IV | NR | NR | NR | NR | NR | NR | Almost all the parents of the patients diagnosed with inborn error of metabolism were consanguineous. | NR |
| Lin 2009  Taiwan[^15^](#_ENREF_15) | MPS IVA  MPS IVB | NR | NR | NR | NR | NR | NR | NR | NR |
| Al-Jasmi 2010  UAE[^16^](#_ENREF_16) | MPS IVA | NR | NR | NR | 2 patients in 1/26 Emiratis tribes (tribe H) had a mutation = c.319G>A in the GALNS gene. No data for non-emirates |  | 50% UAE (n=2);  50% NR (n=2 expatriates) | The UAE local society remains tribal in nature and consists of at least 70 distinct tribes. Despite this ethnic diversity, inter-tribal marriages are less common than intra-tribal ones. Thus, the culture enforces appearance of rare recessive conditions. | NR |
| Nelson 1997  UK (NI)[^17^](#_ENREF_17) | MPS IVA  MPS IVB | NR | NR | NR | NR | NR | NR | NR | NR |
| KOL UK[^18^](#_ENREF_18) | MPS IVA  MPSIVB | 52 | 15.4 (mean) | 23kg (mean weight) | NR | Ethnicity founder effect in Pakistani population | NR (live in UK) | NR | NR |

Abbreviations: BC=British Columbia; B&C= Boyacá and Cundinamarca; EP=Eastern Province; KOL=Key opinion leader; MPS = mucopolysaccharidoses; NI=Northern Ireland; Not recorded; POR=Patient organisation representative; UAE= United Arab Emirates; UK=United Kingdom; W=West.

[1] Fietz M. MPS IV Prevalence (Australia) [Personal communication]. 19/03/2014.

[2] Meikle PJ, Hopwood JJ, Clague AE, Carey WF. Prevalence of lysosomal storage disorders. *JAMA* 1999;281(3):249-54.

[3] Nelson J, Crowhurst J, Carey B, Greed L. Incidence of the mucopolysaccharidoses in Western Australia. *Am J Med Genet* 2003;123 A(3):310-313.

[4] Giugliani R. MPS IV prevalence (Brazil) [Personal communication]. 20/01/2014.

[5] Applegarth DA, Toone JR, Lowry RB. Incidence of inborn errors of metabolism in British Columbia, 1969-1996. *Pediatrics* 2000;105(1):e10.

[6] Lowry RB, Renwick DH. Relative frequency of the Hurler and Hunter syndromes. *N Engl J Med* 1971;284(4):221-2.

[7] Lowry RB, Applegarth DA, Toone JR, MacDonald E, Thunem NY. An update on the frequency of mucopolysaccharide syndromes in British Columbia. *Hum Genet* 1990;85(3):389-90.

[8] Gomez AM, Garcia-Robles R, Suarez-Obando F. [Estimation of the mucopolysaccharidoses frequencies and cluster analysis in the Colombian provinces of Cundinamarca and Boyaca]. *Biomedica* 2012;32(4):602-9.

[9] Malm G, Lund AM, Mansson JE, Heiberg A. Mucopolysaccharidoses in the Scandinavian countries: incidence and prevalence. *Acta Paediatr* 2008;97(11):1577-1581.

[10] Baehner F, Schmiedeskamp C, Krummenauer F, Miebach E, Bajbouj M, Whybra C, et al. Cumulative incidence rates of the mucopolysaccharidoses in Germany. *J Inherit Metab Dis* 2005;28(6):1011-7.

[11] Akiyama T. MPS IV prevalence (Japan) [Personal communication]. 17/01/2014.

[12] Lock Hock N. MPS IV Prevalence (Malaysia) [Personal communication]. 14/03/2014.

[13] Poorthuis BJHM, Wevers RA, Kleijer WJ, Groener JEM, De Jong JGN, Van Weely S, et al. The frequency of lysosomal storage diseases in the Netherlands. *Hum Genet* 1999;105(1-2):151-156.

[14] Moammar H, Cheriyan G, Mathew R, Al-Sannaa N. Incidence and patterns of inborn errors of metabolism in the Eastern Province of Saudi Arabia, 1983-2008. *Ann Saudi Med* 2010;30(4):271-7.

[15] Lin HY, Lin SP, Chuang CK, Niu DM, Chen MR, Tsai FJ, et al. Incidence of the mucopolysaccharidoses in Taiwan, 1984-2004. *Am J Med Genet A* 2009;149(5):960-964.

[16] Al-Jasmi FA, Tawfig N, Berniah A, Ali BR, Taleb M, Hertecant JL, et al. Prevalence and novel mutations of lysosomal storage disorders in United Arab Emirates : LSD in UAE. *JIMD Rep* 2013;10:1-9.

[17] Nelson J. Incidence of the mucopolysaccharidoses in Northern Ireland. *Hum Genet* 1997;101(3):355-358.

[18] Hendriksz CJ. MPS IV prevalence (UK) [Personal communication]. 7/01/2014.
