# Supplementary material for: A systematic review of the prevalence of Morquio A syndrome: challenges for study reporting in rare diseases
Source: Orphanet J Rare Dis. 2014 Nov 18;9:173. doi: 10.1186/s13023-014-0173-x (PMC4251694; doi:10.1186/s13023-014-0173-x)
Supplement: Additional file 6: — Reported study limits. [file 13023_2014_173_MOESM6_ESM.docx]

**Additional file 6: REPORTED STUDY LIMITS FOR ALL COUNTRIES**

| **First author and publication year** | **Country** | **Subgroup** | **Reported limits of the study** |
| --- | --- | --- | --- |
| Meikle 1999[^1^](#_ENREF_1) | Australia | MPS IVA | To calculate incidence, prevalence, and carrier frequency values, we needed to make certain assumptions. We assumed that the rate of postnatal diagnosis was equivalent to the birth rate for each disorder. If the postnatal diagnosis was less than the birth rate, as a result of undiagnosed early death, then our estimates of incidence values would be low. This is unlikely because all unexpected child deaths in Australia result in postmortem examinations, including histopathology studies. We also assumed that the parents of affected individuals were heterozygous for the disorders (with the exception of X-linked disorders). If this were not the case, then our estimates of carrier frequency would be low; however, homozygous parents, in what are predominantly childhood disorders, are not common and, as such, would have little effect on our carrier frequency estimates. For some disorders the number of diagnoses was not high enough to make data statistically significant. No data available on ethnic background. It is possible individuals at less severe end of spectrum may not be diagnosed. |
| Nelson 2003[^2^](#_ENREF_2) | Australia (W) | MPS IVA  MPS IVB | It is possible that some cases may have been born in WA and subsequently left the State to be diagnosed elsewhere, but since migration from the State is small and the population is relatively stable, we feel it is unlikely that a significant number of cases have been lost to ascertainment in this way. No atypical MPS IVA patients were identified in WA. |
| Applegarth 2000[^3^](#_ENREF_3) | Canada (BC) | MPS IVA | Any attempt at generating accurate prevalence or incidence figures based on years of birth will always be subject to problems of underdiagnosis because there will always be some patients who present with signs or symptoms of their inborn disease later in life than the time period surveyed. There is also the aspect to consider of affected infants who move out of the Province before diagnosis. Before 1991, the majority of British Columbians were of British or European origin. According to census Canada the British group comprised 58%, whereas other European populations comprised 29%. Chinese at that time comprised 2% of the population, North American Indians 2.4%, East Indians 0.9%, and Japanese 0.6%. Since 1991 there has been a significant influx of people of Chinese and Indo-Pakistani origin. Ethnicity was not specifically investigated. |
| Lowry 1971[^4^](#_ENREF_4) | Canada (BC) | MPS IV | States 'It is likely that there is an under ascertainment for the last 2 years of the study. It is unlikely that patients 4 or older have been missed since the phenotype becomes so striking' 'although the birth figures ended in 1968, case finding continued until the present' |
| Gomez 2012[^5^](#_ENREF_5) | Colombia (B&C) | MPS IV | Studies to estimate the frequency of MPS have inherent difficulties; there is no mandatory reporting, no system record of this type of disease, no neonatal screening program. There may be underdiagnosis due to; lack of medical staff trained for diagnosis, large variability of clinical manifestations in affected patients, difficult diagnosis. There is no referral center for the diagnosis and management of these diseases, so there is a great number of different institutions responsible for these patients and is extremely difficult access to all records. |
| Malm 2008[^6^](#_ENREF_6) | Denmark | MPS IV | None of the Scandinavian countries employ neonatal screening for mucopolysaccharidoses has so far been performed, and our data rely on the clinical recognition of those rare disorders and performance of relevant laboratory analyses. Milder phenotypes are probably overlooked. In both Denmark and Norway the incidence of Morquio’s disease is higher than that in Sweden, which in both countries is associated with, for the most part, by immigration of Pakistani families. |
| Poorthuis 1999[^7^](#_ENREF_7) | Netherlands | MPS IVA  MPS IVB | The age of diagnosis in the adult patient group varied from 11–64 years of age suggesting great variation in clinical severity extending into a very mild phenotype expressing itself at an advanced age. Indeed, the very mild phenotype may go unrecognized. Patients with very mild complaints may be under-represented in this survey. |
| Lin 2009[^8^](#_ENREF_8) | Taiwan | MPS IVA  MPS IVB | we recognize that few patients could be missed are ‘‘less coarse,’’ but may be very ill from cardiovascular and respiratory complications in infancy; therefore, not recognized by primary care providers and died young before the diagnosis was made. |
| Nelson 1997[^9^](#_ENREF_9) | UK (NI) | MPS IVA  MPS IVB | Complete ascertainment of MPSIVA is unlikely as several mild or atypical patients may have been undiagnosed. The incidence rate of MPSIVA is considerably higher than the incidence in the British Columbian population. It is probable that the British Columbia data comprise mainly severe “classical” cases of Morquio syndrome.  Since patients with MPSIVB are said to be clinically milder than cases of MPSIVA it may be that some of these cases were undetected, although MPSIVB would almost certainly be much rarer than MPS IVA in the Northern Ireland population. |
| Al-Jasmi 2010[^10^](#_ENREF_10) | UAE | MPS IVA | In this series, most of the recognized cases of LSD are of the severe infantile form, while the treatable adult forms are less frequent. The severe forms of LSD are easily recognized by their striking features, while the milder forms are easily missed. Therefore, it is likely that the milder forms of LSD are under diagnosed in UAE. |

Abbreviations: BC=British Columbia; B&C= Boyacá and Cundinamarca; GAG= Glycosaminoglycans, KS= Keratin Sulphate, LSD= Lysosomal Storage Dieases, MPS IV = Mucopolysaccharidoses Type 4, MPS IVA = Mucopolysaccharidoses Type 4A, MPSIVB = Mucopolysaccharidoses Type 4B, NI=Northern Ireland; UAE=United Arab Emirates, UK=United Kingdom; USA=United States of America, USSR=Union of Soviet Socialists Republics

[1] Meikle PJ, Hopwood JJ, Clague AE, Carey WF. Prevalence of lysosomal storage disorders. *JAMA* 1999;281(3):249-54.

[2] Nelson J, Crowhurst J, Carey B, Greed L. Incidence of the mucopolysaccharidoses in Western Australia. *Am J Med Genet* 2003;123 A(3):310-313.

[3] Applegarth DA, Toone JR, Lowry RB. Incidence of inborn errors of metabolism in British Columbia, 1969-1996. *Pediatrics* 2000;105(1):e10.

[4] Lowry RB, Renwick DH. Relative frequency of the Hurler and Hunter syndromes. *N Engl J Med* 1971;284(4):221-2.

[5] Gomez AM, Garcia-Robles R, Suarez-Obando F. [Estimation of the mucopolysaccharidoses frequencies and cluster analysis in the Colombian provinces of Cundinamarca and Boyaca]. *Biomedica* 2012;32(4):602-9.

[6] Malm G, Lund AM, Mansson JE, Heiberg A. Mucopolysaccharidoses in the Scandinavian countries: incidence and prevalence. *Acta Paediatr* 2008;97(11):1577-1581.

[7] Poorthuis BJHM, Wevers RA, Kleijer WJ, Groener JEM, De Jong JGN, Van Weely S, et al. The frequency of lysosomal storage diseases in the Netherlands. *Hum Genet* 1999;105(1-2):151-156.

[8] Lin HY, Lin SP, Chuang CK, Niu DM, Chen MR, Tsai FJ, et al. Incidence of the mucopolysaccharidoses in Taiwan, 1984-2004. *Am J Med Genet A* 2009;149(5):960-964.

[9] Nelson J. Incidence of the mucopolysaccharidoses in Northern Ireland. *Hum Genet* 1997;101(3):355-358.

[10] Al-Jasmi FA, Tawfig N, Berniah A, Ali BR, Taleb M, Hertecant JL, et al. Prevalence and novel mutations of lysosomal storage disorders in United Arab Emirates : LSD in UAE. *JIMD Rep* 2013;10:1-9.
